# A Rare Case of Paratesticular Leiomyosarcoma

**DOI:** 10.7759/cureus.43294

**Published:** 2023-08-10

**Authors:** Ernesto O Munoz Pena, Keishla Garcia Fernandez, Abigail Miller, Jonathan Vasquez, Vania Zayat

**Affiliations:** 1 Internal Medicine, University of Central Florida HCA Healthcare GME, Greater Orlando, Orlando, USA; 2 Pathology, University of Central Florida College of Medicine, Orlando, USA; 3 Pathology, Orlando Veterans Affairs Medical Center, Orlando, USA

**Keywords:** paratesticular leiomyosarcoma, paratesticular mass, soft tissue tumours, primary leiomyosarcoma, para testicular tumors

## Abstract

Paratesticular leiomyosarcoma is a rare urologic cancer that arises from undifferentiated smooth muscles of the spermatic cord or epididymis. Few accounts of this cancer have been reported but previous reports have identified radiation and anabolic steroids as possible risk factors. We report a case of an 83-year-old man with a previous history of radiation therapy for prostate cancer, who presented with a painless left scrotal mass. Given the nonspecific presentation, a histopathological classification was warranted for a definitive diagnosis. The tumor was resected via simple orchiectomy and was diagnosed as a paratesticular grade III leiomyosarcoma without any further treatment. Patient had a follow-up CT scan of the abdomen and pelvis that was normal without metastasis. The patient’s history of previous external beam radiation and now development of a secondary tumor sums to the few cases that have been previously reported with this association.

## Introduction

Paratesticular leiomyosarcoma is a malignant soft-tissue tumor that arises from undifferentiated smooth muscle cells most commonly originating from the spermatic cord and the epididymis [1,2]. It is considered a rare cause of paratesticular tumor that presents as a slow-growing solid scrotal mass that cannot be differentiated from other tumors unless histological evaluation is performed [3]. Presently, only a limited number of cases can be found in the literature. Here, we present a rare case of an elderly patient with paratesticular leiomyosarcoma presenting with left scrotal mass. In this report, we discuss the risk factors, diagnostic, and management approach for this rare tumor. 

## Case presentation

An 83-year-old man with past medical history of prostate cancer diagnosed nine years ago and treated with external beam radiation followed by hormonal therapy presented to the primary care provider complaining of a left scrotal swelling. The swelling appeared one or two years prior to presentation but got progressively worse a few months before the visit. The patient denied scrotal pain, redness, urgency, frequency, dysuria or discharge. Family history was remarkable for prostate cancer in his father. On physical examination there was a left scrotal swelling that was non-tender or erythematous and no paratesticular mass was palpable. Right testis was unremarkable. 

**Figure 1:**
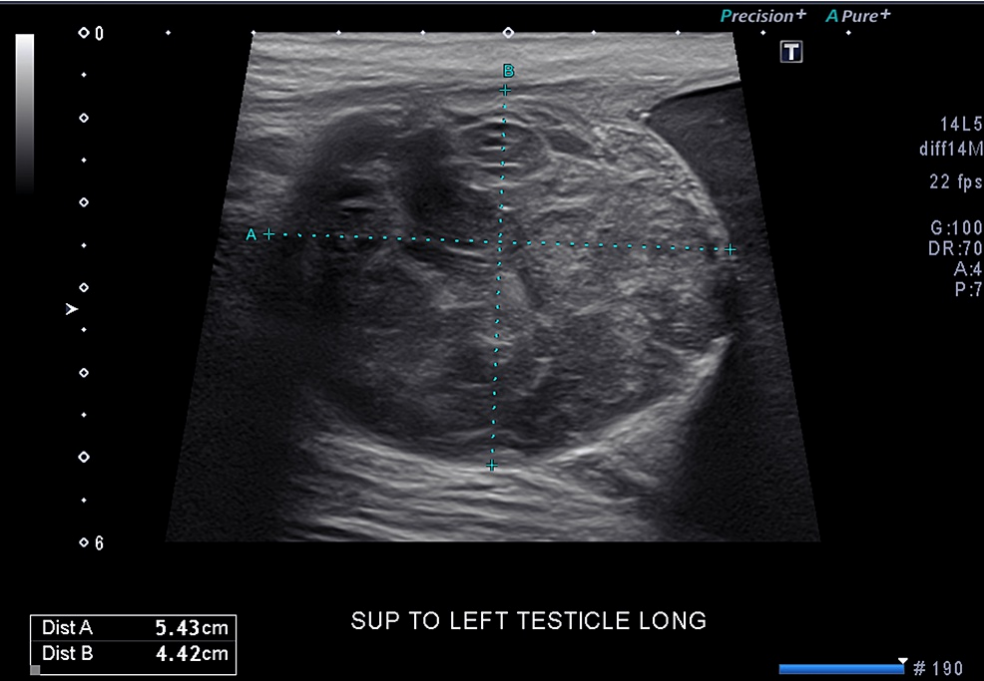
Scrotal ultrasound showing a well-defined hypoechoic paratesticular mass located in the superior aspect of the left testicle.

Laboratory findings, including complete blood cell count, complete metabolic panel and urinalysis were unremarkable. Prostate-specific antigen (PSA) was normal for the last eight years. Patient’s tumor markers, which included beta-human chorionic gonadotropin (beta-hCG), alpha-fetoprotein (AFP) and lactate dehydrogenase (LDH) were within normal limits. Scrotal ultrasound revealed normal-sized testicles with a well-defined hypoechoic paratesticular mass located in the superior aspect of the left testicle with internal hypervascularity measuring 4.4 x 4.2 x 5.4 cm (Figure 1). There was also an enlarging hydrocele in the left hemiscrotum containing homogeneous internal echoes displacing the left testis peripherally. 

**Figure 2:**
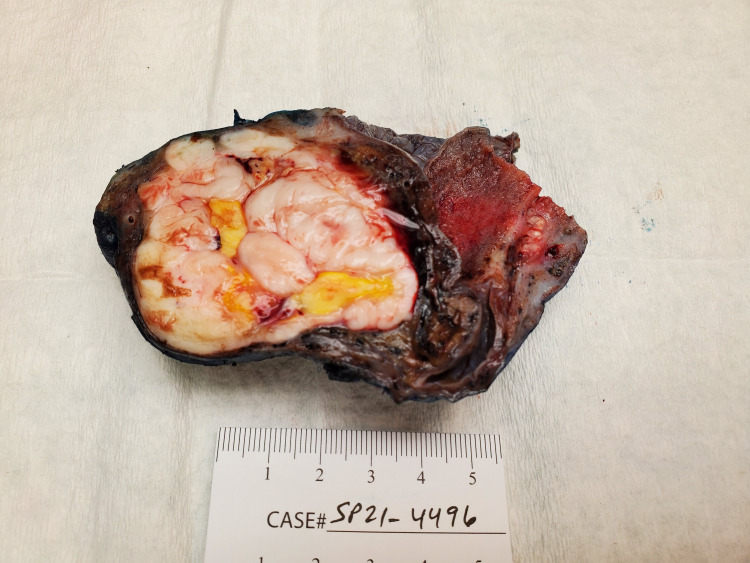
Gross pathology of left paratesticular scrotal mass.

The operative findings were a left hydrocele, atrophic left testis and solid left paratesticular scrotal mass superior to the testicle compromising the vasculature to the left testicle. Left simple orchiectomy with excision of paratesticular scrotal mass and hydrocele were performed. Gross pathology of the specimen (labeled as left hydrocele, testicle and scrotal mass) revealed a tan, purple rubbery to firm, semi-saccular tissue, measuring 11.5 x 6.2 x 5.0 cm. The specimen margin was inked in blue and bivalved revealing a circumscribed, firm mass, measuring 8.0 x 5.0 x 4.8 cm. The mass displayed lobulated pale tan-white to focally yellow cut surfaces with scattered necrotic foci (Figure 2).

**Figure 3:**
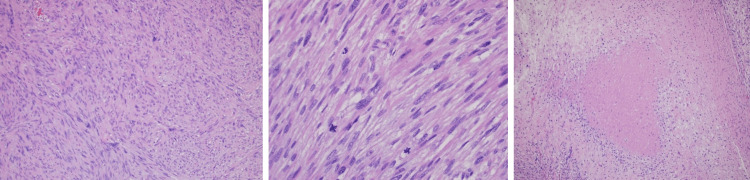
Left panel showing a low power view of whorled pattern with spindle cells and nuclear pleomorphism. Middle panel showing histopathology demonstrating mitosis and right panel with necrosis.

**Figure 4:**
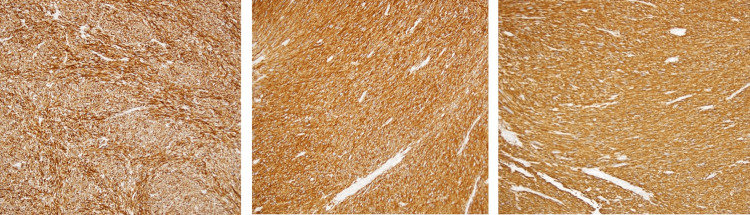
Immunohistochemistry slides: Left panel positive h-caldesmon; middle panel positive desmin; right panel positive SMM-HC

**Figure 5:**
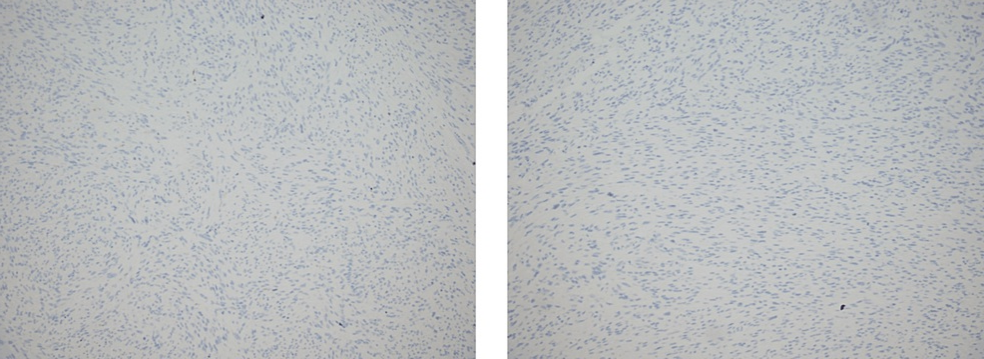
Immunohistochemistry slides: Left panel negative S-100; right panel negative calretinin.

Upon microscopic evaluation, the mass was composed of intersecting, sharply marginated fascicles of spindle cells with elongated, hyperchromatic nuclei, and abundant eosinophilic cytoplasm (Figure 3). There were 20-25 mitotic figures per high power field (HPF) and necrosis was present in 15% of the sample (Figure 3). Immunohistochemical staining was positive for desmin, smooth muscle myosin heavy chain (SMM-HC), smooth muscle actin (SMA), and h-caldesmon (Figure 4). It was negative for S-100, CD 117, and calretinin (Figure 5). High levels of Ki67 (40%) were noted. Final diagnosis based on histopathological and immunohistochemical examinations of the mass was characteristic of grade III leiomyosarcoma. Margins were negative and no lymphovascular invasion was identified. Surveillance computerized tomography (CT) scan of the abdomen and pelvis, performed three months after left orchiectomy, revealed no evidence of metastatic disease or adenopathy. Patient did not receive radiation or chemotherapy after orchiectomy. Patient decided not to follow up with oncology.

## Discussion

Intrascrotal tumors are categorized into testicular and paratesticular tumors. Paratesticular tumors account for 7 to 10% of all intrascrotal tumors with 30% of these tumors being malignant [1,4]. Most common types of paratesticular tumors are liposarcomas, leiomyosarcomas, rhabdomyosarcomas and fibrosarcomas [1]. Leiomyosarcoma, which accounts for approximately 20% of the paratesticular tumors, is a malignant mesenchymal tumor arising from smooth muscle cells located in the epididymis, vas deferens, cremasteric muscle, tunica testis or the spermatic cord, the last being the most common place of origin [4]. This is a rare tumor that has been described in the literature with a little more than 200 cases [5].

Paratesticular leiomyosarcomas usually affect elderly patients in the sixth to seventh decade of life although cases have been reported in younger male adults [1]. Paratesticular leiomyosarcomas most commonly present as a painless, slow-growing, palpable solid scrotal mass, sometimes associated with hydrocele [1,4]. There have been case reports suggesting that development of these tumors is associated with anabolic steroid use or radiation exposure as predisposing factors [6,7]. This is an interesting fact because our patient received external beam radiation and hormonal therapy as part of his prostate cancer treatment which could be the initiating factor for development of the leiomyosarcoma.

Tumor markers like beta-hCG, LDH and AFP are usually negative. Tumor markers in our patient were all negative. Ultrasound is the initial imaging of choice for evaluation of any intrascrotal mass. Leiomyosarcomas can appear on ultrasound as a heterogenous lesion but can also appear as a hypoechoic lesion. CT/MRI can help to differentiate the lesion, but definite diagnosis is made by histological evaluation [1,4]. Our patient had an orchiectomy with histology showing spindle cells in whorled pattern with atypia, mitoses, and necrosis. Immunohistochemistry usually stains positive for smooth muscle actin, muscle-specific actin, h-caldesmon and desmin, indicating smooth muscle differentiation. Immunohistochemical staining in our patient was positive for desmin, SMM-HC, SMA, and h-caldesmon (Figure 4). It was negative for S-100, CD 117, and calretinin (Figure 5). High levels of Ki67 (40%) were noted. The most reliable histological indicator of malignancy is mitotic index (MI or mitotic figures per 10 HPF). A mitotic index > 10 indicates malignancy. Our case had 20-25 mitotic figures per high power field and 15% tumor necrosis. Therefore, the tumor was classified as a grade III tumor using the NCI grading system. Grading of the tumor based on histology is important to determine prognosis. High-grade tumors are more aggressive [1].

Radical orchidectomy with high ligation of the spermatic cord is the standard treatment for these tumors [1,5,8]. There is insufficient data on prophylactic lymph node dissection for relapse prevention. Although there are studies that may suggest that adjuvant radiotherapy may be effective in treating loco-regional microscopic disease [9], it has not shown survival benefit. Chemotherapy is controversial and has only been used in the case of metastatic disease [1,4]. Although there is limited data about this tumor, some literature mentioned that five- and 10-year disease-specific survival rates are 77% and 66% respectively [10]. Regardless of this relatively good long-term survival, because recurrences are common, close follow-up is suggested for disease surveillance.

## Conclusions

Paratesticular leiomyosarcoma is a rare type of paratesticular tumor that has been associated with previous radiation therapy in a few cases in the literature. Due to the rarity of this lesion and a broad differential diagnosis of testicular masses, it is commonly misdiagnosed before histological examination. Therefore, histopathology evaluation is the goal standard for definitive diagnosis of leiomyosarcoma since it cannot be diagnosed based on physical exam or laboratory findings. Our patient’s history of previous external beam radiation and now development of a secondary tumor sums to the few cases that have been previously reported with this association.
